# Channel State Estimation in LTE-Based Heterogenous Networks Using Deep Learning

**DOI:** 10.3390/s21227716

**Published:** 2021-11-19

**Authors:** Krzysztof K. Cwalina, Piotr Rajchowski, Alicja Olejniczak, Olga Błaszkiewicz, Robert Burczyk

**Affiliations:** Faculty of Electronics, Telecommunications and Informatics, Gdansk University of Technology, 80-233 Gdansk, Poland; piorajch@eti.pg.edu.pl (P.R.); alicja.olejniczak@pg.edu.pl (A.O.); olga.blaszkiewicz@pg.edu.pl (O.B.); robert.burczyk@pg.edu.pl (R.B.)

**Keywords:** deep learning, heterogeneous network, channel state, LTE

## Abstract

Following the continuous development of the information technology, the concept of dense urban networks has evolved as well. The powerful tools, like machine learning, break new ground in smart network and interface design. In this paper the concept of using deep learning for estimating the radio channel parameters of the LTE (Long Term Evolution) radio interface is presented. It was proved that the deep learning approach provides a significant gain (almost 40%) with 10.7% compared to the linear model with the lowest RMSE (Root Mean Squared Error) 17.01%. The solution can be adopted as a part of the data allocation algorithm implemented in the telemetry devices equipped with the 4G radio interface, or, after the adjustment, the NB-IoT (Narrowband Internet of Things), to maximize the reliability of the services in harsh indoor or urban environments. Presented results also prove the existence of the inverse proportional dependence between the number of hidden layers and the number of historical samples in terms of the obtained RMSE. The increase of the historical data memory allows using models with fewer hidden layers while maintaining a comparable RMSE value for each scenario, which reduces the total computational cost.

## 1. Introduction

Modern radio communication must meet a few fundamental requirements: availability, user-oriented services, and reliability. These assumptions may be combined into one goal—creation of a multi-functional network. The new and next generation networks, like LTE (Long Term Evolution) and 5G, can be adopted to create a flexible heterogeneous environment, also by using the SDR (Software Defined Radio) technology [1]. Such a concept assumes the coexistence of at least two RIs (Radio Interfaces) in the UE (User Equipment), so-called multi-link. In fact, the heterogeneity should be considered not only as a presence of two different technologies. The presence of two different cells (e.g., the macro and nano cells) or two different networks operating at the same area meet the mentioned criteria as well.

The network heterogeneity implies the extensive changes in the UE software. If the communication can be realized by two independent RIs, the decision must be made in terms of how to organize the data transmission, minimizing e.g., BLER (Block Error Rate), especially if the data are assigned to different services [2,3]. Henceforth, the effectiveness of the radio link must be introduced. In general, when the UE generates data with a different priority, the decision process of the DAA (Data Allocation Algorithm) should involve the states of the RIs to allow estimating the cost of the transfer—probability of loss of the information—with an assigned priority. Hence, the analysis of the past and current RIs states, e.g., the physical layer quality parameters both within “active transmission” and “idle” states, may be essential in terms of general DAA effectiveness.

The goal of the conducted research was to develop a method that will be the part of the DAA, and will provide a high efficiency of the transmission quality prediction on the basis of the measured radio channel parameters in the heterogeneous cellular networks. Based on the measurement methodology, commercial radio module that has been used and the presented DL (Deep Learning) approach, the proposed DAA should be considered as innovative relative to the state-of-the-art research in a given area. Furthermore, the proposed approach has a great utility value in the context of the real applications and it is a response to the market needs.

As it has been mentioned, the heterogeneous node has the ability to transmit selected data streams through multiple RIs. Thus, the decision to choose the exact communication interface for the next successful transmission is treated as the conditional probability of the transmission with the certain parameters (e.g., BER - Bit Error Rate, BLER, delay, etc.) under certain propagation conditions. Propagation conditions determine the quality of the received signal, which parameters are estimated by the mobile terminals and the base stations of a given network, and are expressed parametrically by e.g., RSSI (Received Signal Strength Indicator), SNR (Signal to Noise Ratio), SINR (Signal to Interference and Noise Ratio), etc. These quality indicators can be estimated during the transmission and stored by the node for further decision making regarding the next transmission, before it is actually done. In the paper, the DL-based implementation of the estimation mechanism is presented, what may be treated as an innovative approach regarding non-linear modelling of the parameters transfer function in that case. It can be used for an effective selection of the communication link in the heterogeneous telemetry device in the scope of prediction of the selected metrics for assessing the quality of the radio link.

The rest of the paper is structured as follows. In the Section 2 the related works regarding the DL and heterogeneous networks are described. In the Section 3 the concept of the data allocation algorithm is presented. The Section 4 contains the description of the measurement stand, and in the Section 5 the measurement scenarios are presented. In the Section 6 authors have presented the preliminary results of the conducted research studies for the link state estimation. In the Section 7 the DL model is presented as an alternative for the typical approach. The Section 8 summarizes the paper.

## 2. Related Work

The DAAs are widely analyzed and discussed in the context of the heterogeneous networks [4,5,6]. This problem is often connected with the throughput profiling [7] or the IoT (Internet of Things) and 5G networks [8,9]. In [7] authors proposed a solution for a dense industrial IoT network where many influencing to each other cells may be distinguished. It was pointed that the proposed framework [9] can be improved by the DL what will lead to a better accuracy of the RI state estimation.

Generally, the DL support is used due to its effectiveness in terms of solving the non-linear problems, which is desirable in the realm of wireless communication as well [10,11]. The concept of the 5G network assumes the existence of many terminals at the same area [12]. Moreover, the mobility of users is the essential part of radio resource utilization algorithms that introduces a stochastic character of the input data. In [13] authors proposed to use the deep neural network to capture the information about the network utilization (considering the behavior of the users) and further adapt the TDD (Time Division Duplex) uplink/downlink organization. Conducted simulation studies [13] showed that it was possible to increase the network performance, the available throughput, and decrease the packet loss rate. Consequently, the quality of service can be noted as better for the end user, nevertheless in this example the network remains non-heterogeneous.

In the modern networks the available throughput may be increased not only by extending the bandwidth, but also by creating the coordinated multi-point architecture [14]. In this case, besides the macro cells, the smaller micro cells were deployed on the same area, which implies the heterogeneous structure of the network and allows the terminal to transmit the data using the two RIs with statistically independent propagation conditions. In the paper [14] authors improved the performance of the OFDM (Orthogonal Frequency Division Multiplexing) based 5G network by implementing the SINR and RSRP (Reference Symbol Received Power) as input parameters for the deep neural network managing the data streams. The presented results showed that DL-based approach allowed for increasing the downlink throughput. Unfortunately, authors of [14] did not include in the analysis other physical layer quality parameters (described in [15,16]) and they did not assume increasing the reliability of the radio link.

Other examples of DL application in the OFDM-based network are the channel influence mitigation [17,18] and the anomalies detection. In [19] authors proposed a method for detecting the unexpected traffic fluctuations in the 5G network. To make the decision process reliable the two-level DL model was proposed. The division of the model is implied by the network architecture. The proposed DL-based framework may be implemented to support the RAN (Radio Access Network) architecture to detect characteristic symptoms of the cyberattack. The master layer of the model is based on the LSTM (Long Short-Term Memory) recurrent neural network that is used to judge the potential anomalies and recognize the patterns of the cyberattack. The method presented in [19] detects the RAN state effectively; nevertheless, the main goal of the presented research is to inspect the operation of the network and make it resistant to intentional attacks. Moreover, there is no possibility to adjust the operation of the user terminals, e.g., select the RI, depending on the state of the network.

Data allocation algorithms presented in the literature are mainly focused on maximizing the throughput offered to the user. Nevertheless, in some applications, like the telemetry of the critical infrastructure, the most important aspect is the reliability. The measurement data or alarm flags may be treated as data with various priorities and different frequency of occurrence. Thus, it influences the operation and design of the algorithms, mainly the estimation of the heterogeneous RIs parameters. Taking this issue into consideration, authors proposed the DL-based channel state estimation method dedicated for special applications that assumes the usage of the LTE RI. The presented solution provides higher effectiveness regarding the linear model for channel state metrics estimation. Undoubtedly, the considered issue of determining and predicting the channel state estimates (and as a result the transmission quality) based on the set of parameters is a non-linear dependency, which should be deeply investigated especially in the heterogeneous networks. The conducted research has proved that the analyzed wide set of parameters, measured before the transmission by the commercial radio module in the LTE RI combined with the further usage of the proposed DL-based methods enabled providing high accuracy of the BLER prediction, which was assumed as the main transmission quality metric.

## 3. Data Allocation Algorithm

**Figure 1:**
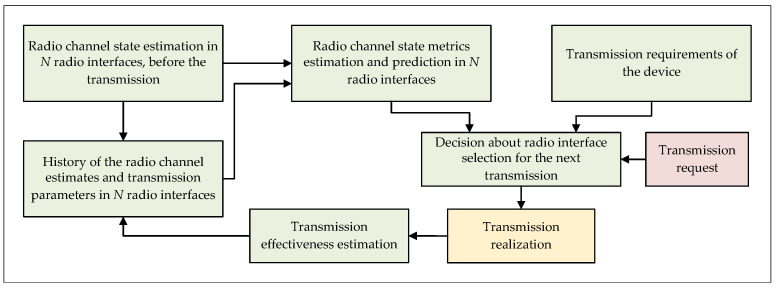
The block diagram of the data allocation algorithm for selecting the optimal radio link in the heterogeneous node.

Undoubtedly, the data allocation algorithm must take into account not only the operating characteristics of the RIs, but also make a decision on the selection of a given interface based on the additional requirements and the operational parameters of a given telemetry device, e.g., total delay in the point-to-point relation, or the cost of transmission (e.g., financial cost, RI delay, energy consumption, etc.). Considering previously mentioned issues, the algorithm for selecting the optimal radio link for the next transmission in a heterogeneous node is shown as a block diagram in the Figure 1 and as a pseudocode in the Algorithm 1.

For the implementation of the DAA that routes packets with the user data, it is necessary to determine the measurable quality parameters of the radio channel before the next transmission will be carried out. However, it should be remembered that the set of parameters that can be estimated is limited, and generally defined by the radio modem used as the RI in the heterogeneous node. The determined radio channel quality estimates (the radio channel parameters) provide useful information not only for the currently planned transmission, but also to some extent for the subsequent ones, while the length of the history of the analyzed samples should be determined through the measurement tests and deep analysis, which was done in the conducted research studies and is described further in the article.
**Algorithm 1** Pseudocode of the data allocation algorithm.**Require:** 
Transmission requirements;**Ensure:** 
Estimates of transmission effectiveness (BLER); Parameters of N radio interfaces;**while***true***do**State estimation in *N* radio interfaces before the transmission;Update and predict channel state metric of *N* radio interfaces;    **if** *Transmission request* **then**        Select the radio interface;        Realize transmission;        Estimate the transmission effectiveness;        Update the historical metrics of the radio channel estimates;        Update the historical metrics of the transmission        parameters in *N* radio interfaces;    **end if****end while**

The estimation and prediction of the available RIs quality metrics can be performed in many ways, but in general we can distinguish the linear and non-linear methods. It appears that the mapping of the analyzed channel quality estimates and the prediction of the selected quality metrics for the individual RIs should be a non-linear function. Moreover, the linear function will enable one to determine the lower bound of the accepted solutions in that case. The upper theoretical bound is the possibility of predicting the quality metrics of RIs with the probability equal to 1. The quality metrics can be both the parameters defining the uplink or downlink radio link parameters (e.g., BER, BLER) and transmission parameters depending on the characteristics of the mobile operator’s network (e.g., transmission delay at the level of the IP layer). The selection of the quality metrics for the RIs is extremely important, especially in the context of the operation efficiency (the effectiveness of the decision-making process) of the DAA for different RIs operating within various radiocommunication systems (GSM - Global System for Mobile Communications, UMTS - Universal Mobile Telecommunications System, LTE etc.). This resolves the method of analyzing the individual metrics for the several interfaces and forces the estimation of their transfer function determining the probability of correct transmission with selected communication parameters.

After the data allocation decision-making process is completed, based on the predicted metrics of the radio channel quality (or transmission quality) and the defined functional requirements of the telemetry device, the transmission efficiency is estimated. It is defined as the error estimation of the expected values with respect to the actual values obtained during the transmission of the user data. These parameters are transferred to the memory block storing the data history with a defined memory length, which will be updated during the device operation and used to increase the efficiency of the subsequent transmissions.

**Figure 2:**
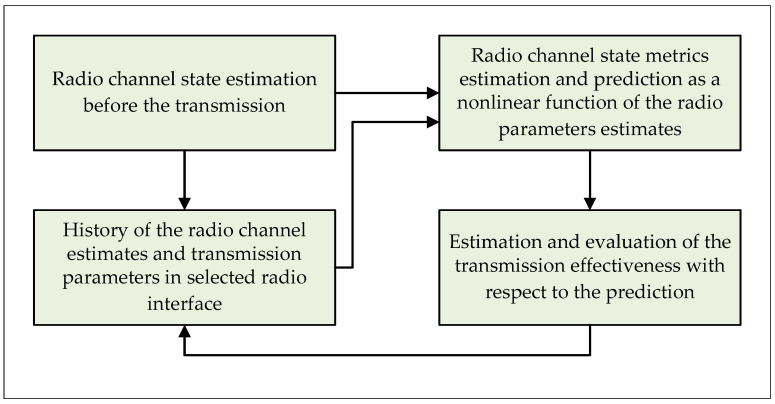
The block diagram of the decomposed DAA algorithm for selecting the optimal radio link.

Undoubtedly, the final quality of the DAA algorithm performance is determined by the efficiency of estimation and prediction of the quality parameters for each RI separately. Considering the above, it is possible to decompose the communication link selection algorithm presented in the Figure 1 for separate RIs and determine the effectiveness of its operation. In the Figure 2 and Algorithm 2 the block diagram and pseudocode of the decomposed DAA algorithm for each RI are shown.
**Algorithm 2** Pseudocode of the decomposed data allocation algorithm.**Require:** 
Number of the radio interface;**Ensure:** 
Estimates of transmission effectiveness (BLER); Parameters of the radio interface;Radio channel state estimation before the transmission;**while***true***do**    Update channel state metrics of the radio interface;    Predict channel state metrics of the radio interface;    Estimate the transmission effectiveness;    Evaluate the transmission effectiveness;    Update the historical metric of the radio channel estimates;    Update the historical metric of the transmission    parameters of the radio interface;**end while**

In order to conduct research regarding the effectiveness of the DAA algorithm, it is necessary to perform partial research on the selected RIs, separating them from the structure of the entire algorithm. In such case, it is possible to define the operation efficiency of the individual communication interfaces (which differ significantly from each other) and to estimate the transmission quality metric in a given RI that will be analyzed in the final decision-making process of the packet routing in the heterogeneous device. Naturally, the algorithm decomposed for a single RI does not take into account the decision-making process, but only provides the predicted estimates of the transmission quality metric, which is implemented as a non-linear function of the radio parameter estimates. Due to the complicated analysis process of such algorithm, authors decided to split it, and in the presented research studies analyze the LTE RI as the one available RI, where further RIs are a great material for further works in that research field.

The functional module of the transmission quality estimation and evaluation in relation to the predicted parameters requires to carry out the study of the effective method for the implementation of the aforementioned non-linear function. However, it can potentially be an element used in the real operating conditions of the heterogeneous device. This may provide the information about the state and quality of the radio channel in the selected locations of the telemetry devices installation, unique for each telemetry device, and the potential adjustment of the selected method to increase the efficiency of the algorithm for a given RI.

## 4. Measurement Stand

**Figure 3:**
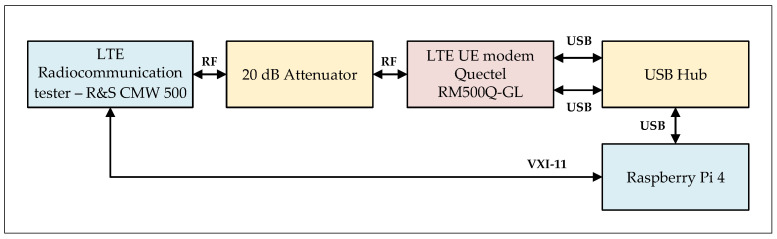
The measurement stand block diagram.

In order to carry out the measurement campaigns, the measurement stand was developed. The prepared measurement stand is described in details in [20,21]. In the Figure 3, the block diagram of the measurement stand is presented.

The CMW500 radiocommunication tester emulates the LTE eNodeB (Evolved Node B), and the RM500Q-GL radio module is used as the UE RI. The single-board computer is a device that allows managing the tester and the radio module using the developed software dedicated for the automatic measurements.

In order to read the parameter’s values, a connection between the UE and the eNodeB is established. Reading the parameters of interest, the communication is carried out in two ways: with the tester by using the SCPI (Standard Commands for Programmable Instruments) commands, and with the module by using the Hayes commands set.

The RF connections between the elements are wired what ensures stable and repeatable propagation conditions (with emulated proper fading profile) and no additional influence of the environment. The additional 20 dB attenuation added in the RF path is needed to provide the acceptable radio signal power at the input of the CMW500. Its value is compensated during the measurements.

## 5. Measurement Scenarios

Two measurement campaigns were carried out as a part of the LTE RI research. The first measurement campaign (MC1) was performed for the operating parameters of the emulated base station presented in the Table 1.

The second measurement campaign (MC2) was carried out mostly for the same parameters of the emulated base station, except that the CQI value was not a parameter defined a priori on the radio tester side, but was determined automatically by the radio tester in accordance with the eNodeB operation method (following the LTE system principles).

It was determined that the analysis in the context of the DAA implementation will focus in particular on the parameters available and obtainable from the radio modules before potential transmission using the LTE RI under the target operating conditions in the real environment. For the implementation of the DAA, the transmission quality parameters and radio channel quality estimates (compliant with the standard) were obtained in the LTE interface, i.e.,:

- BLER [%];
- RSRP before and during transmission;
- RSRQ (Reference Signal Received Quality) before and during transmission;
- RSSI before and during transmission;
- SINR before and during transmission;
- MCS (Modulation and Coding Scheme) before and during transmission.

During the measurements, the variable SNR was obtained by changing the power of the RF signal generated by the tester, not by changing the interference power (the filtered white noise) with respect to the useful signal.

## 6. Experimental Studies

It was established that the transmission quality metric will be the BLER, defined as the number of incorrectly received resource blocks in the PDSCH (Physical Downlink Shared Channel) to the number of all resource blocks sent during the transmission [22]. The BLER calculation is performed on the basis of the calculated CRC (Cyclic Redundancy Code) during the channel decoding in the receiver, i.e., still in the physical layer of the RI. However, it should be noted that the BLER is measured physically by the radio tester not the UE, based on the number of acknowledgments sent by the RI in the uplink, which directly corresponds to the mentioned incorrectly received resource blocks in the PDSCH.

Considering the concept of the DAA described in the Section 3, it was assumed that the BLER quality parameter should be calculated before the transmission, and may be potentially formulated as follows:

(1) BLER=f(RSRP,RSRQ,RSSI,SINR,MCS),

which exact structure should be determined on the basis of the obtained measurement data. This function appears to have a non-linear form—in particular, when the number of these parameters will be increased and the estimated “interface quality” will be expressed by a larger number of parameters, such as delay, bit rate or the estimated cost of the transmission itself.

Due to the large statistical sample set, i.e., over 100,000 obtained measurement data for both measurement campaigns, the dedicated software for visualization and preliminary statistical analysis of the obtained results was developed. This step of the analysis is important, especially in the context of the DL usage and the need of training dataset preparation, validation and the testing processes.

### 6.1. Measurement Campaign No. 1

Firstly, on the basis of the results obtained as a part of the MC1 measurement campaign, it was established whether and which radio parameters should be taken into consideration as the arguments of the given function (also for the historical values), i.e., values before the transmission (i.e., $RSRP_{b}$, $RSRQ_{b}$, $RSSI_{b}$, $SINR_{b}$, $MCS_{b}$) or values during the transmission (i.e., RSRP, RSRQ, RSSI, SINR, MCS). For this reason, the differences in the values of the corresponding parameters during and before the transmission were analyzed.

The differences in the values before and during the transmission are normally distributed, with different values of the standard deviations and almost zero mean values. For the RSRP parameter σ=1.5 dB, for the RSSI parameter σ=2.6 dB, for the SINR parameter σ=1.4 dB, for the RSRQ parameter σ=0.5 dB, and for the MCS parameter σ=3.6. For this reason the information value, from the point of view of the estimation and prediction of the subsequent transmissions, is the same for the values obtained before and during the transmission. At the same time, in the context of the DAA functionality, the usage of the parameters before the transmission is more convenient, e.g., there is no need to perform the transmission and the fact that the interface does not have to be selected for the further transmission of the useful data. As a consequence, the only available metric can be the estimated parameters before the transmission (the so-called probing values).

Considering previously mentioned aspects, it was decided that the arguments of the transmission quality metric function for the LTE interface can only be values obtained before the transmission, including the historical values. In this case, there is a need to update the form of the transmission quality metric function (1)

(2) $$BLER=f({\mathrm{RSRP}}_{b},{\mathrm{RSRQ}}_{b},{\mathrm{RSSI}}_{b},{\mathrm{SINR}}_{b},{\mathrm{MCS}}_{b}),$$

where the input arguments are the value vectors with lengths corresponding to the time windows which are analyzed in the process of predicting the quality of the next transmission.

In the first stage of the research, the correlation degree of the selected arguments of the transmission quality metric function (2), i.e., their mean values, medians and the entire data set, in relation to the resulting value of BLER, was checked. The degree of correlation is understood as the determination of the Pearson correlation coefficient [23]. This coefficient ranges from −1 to 1, where 0 means no data correlation, −1 means inversely proportional data correlation and 1 means the dependence of the data proportional to each other.

In the Table 2 the correlation results between the parameter mean value μ, median value *M* and all values with corresponding BLER values obtained for the six measurement series that determine the set CQI values for a given measurement scenarios are presented. The SNR value was changed by the CMW500 tester emulating the eNodeB station in the range from −3 dB to 30 dB with 1 dB step. It is worth noting that the MCS parameter was not included, due to the forced constant value of the CQI parameter, which directly affects the selection of the appropriate MCS, regardless of the propagation conditions and the determined quality parameters of the channel. Hence, the correlation between the MCS parameter and the BLER metric should not be analyzed at this point, however, undoubtedly this parameter should be taken into account in the final form of the model for the LTE RI in the heterogeneous device [24].

As expected, all correlation values are negative due to the inverse relationship of the selected radio channel quality parameters to the BLER, i.e., the higher the received signal power, the higher probability of obtaining a lower BLER is. The obtained results indicate that for the selected measurements, the analysis and implementation of the DAA algorithm in the adopted form only on the mean values μ or medians *M* of the RSRP, RSRQ, RSSI and SINR parameters should not be performed due to the too low correlation values (i.e., not exceeding than −0.67) of these values relative to the resulting BLER. It suggests that the performed data filtration in that case causes the loss of the essential information about the analyzed parameters and BLER dependence. Presented results show that the highest correlation with the BLER value is obtained for instantaneous values without any filtering method (All).

The results also show that for the most resistant to difficult propagation conditions modulation-code schemes (i.e., for the CQI ≤ 3), no relationship was found between the measured parameters and the obtained error rate, which may suggest that the DAA algorithm cannot operate with the given quality parameters in such cases. These results may indicate that the selected range of the SNR values is too narrow, i.e., the obtained BLERs for transmission should be checked, where the SNR is below the −3 dB. However, for the tested Quectel RM500Q-GL radio module, decreasing the SNR value below −3 dB led to synchronization loss of the radio module with the eNodeB and the disability to perform the data transmission.

The results described so far are based on the analysis of the entire measurement datasets and the validity of using the RSRP, RSRQ, RSSI, and SINR parameters measured before transmission to predict the transmission quality is clearly confirmed. The decision to route packets using a given RI will not be made on the basis of the several thousand measurements, e.g., due to limited memory resources, computing capabilities of the nodes, or the short validity times of a given prediction in the context of changes taking place in the radio channel or even the operator’s network itself. Undoubtedly, the history of changes in the parameters of the radio link may increase the efficiency of the estimation of the current state of the link, but only a limited set of measurement results can be practically implemented. Therefore, it is necessary to define the time interval of *W* previous transmissions from which the data should be taken into account.

At this point, it should be noted that the research analysis concerns the case when the interval between successive measurements is about 300 ms, and the time between the acquired probe values and the actual transmission (and the obtained BLER value) was several ms, which means that the radio channel cannot be considered as stationary between individual measurements. However, according to the target stationary operation scenarios for devices including the DAA functionality, the radio channel should not change drastically in its characteristics, e.g., due to the lack of occurrence of fast fading related to the movement of the device or the movement of other elements in the environment. It also means that the expected channel quality will be valid only for a dozen of milliseconds (which is still more than the channel coherence time) and at that time the decision algorithm should decide about the possible data transmission via the LTE RI. Otherwise, it is necessary to repeat the measurements of the probe values and re-determine the predicted channel quality (if the inertia time of the DAA was for some reason longer than the prediction useful life).

The most direct method of determining the successive estimates for a selected subset of the data (the sample history windows) is to perform the low-pass filtration of the data and adopt the determined value as the predicted value at the next timestamp. The filtration process was carried out as an implementation of the FIR (Finite Impulse Response) filter by determining the so-called moving average filter where each filter tap is multiplied by a 1/W weight and the number of the taps is equal to the length of the analyzed sample window *W*.

For the considered measurements and the channel quality parameters, the correlation of the predicted values with the obtained BLER were analyzed. It should be pointed out that the analysis was conducted considering these parameters as a function of the window length *W*. Basing on the results, it was proved that increasing the history length of analyzed data above 15, does not result in a significant (i.e., greater than 0.02) increase in the correlation coefficient for all the analyzed parameters. It was also confirmed that the results for the CQI = 1 and CQI = 3 are not correlated with the resulting BLER. However, the determined correlation results indicate a greater degree of correlation of the results obtained from a short measurement series before the transmission, than the results from the entire data set—which determines the validity of using the selected approach in the DAA algorithm for the LTE RI, at least for the results of the laboratory measurements for a constant CQI value ⩾ 3 in the range of the tested SNR <−3; 30> dB.

### 6.2. Measurement Campaign No. 2

Having the predefined and optimized operation parameters of the algorithm for the individual arguments of the function determining the transmission quality, the form of the multidimensional function (2) should be determined. Basing on the results presented in the Section 6.1, none of the arguments can be reduced, as it can be used to increase the efficiency of the transmission quality metric prediction. As mentioned, the parameters of the function are not statistically independent variables and it seems that the best (most accurate) estimation of the transmission quality can be obtained for the non-linear function form. This means that by determining the linear dependence of this function, one can define the lower bound of the accepted solutions and adopt it as a reference value for further work on functions mapping the predicted values of the MCS, RSRP, RSRQ, RSSI, and SINR parameters on the transmission quality, in this case defined as the BLER.

In order to make the obtained results reliable and to best highlight the actual conditions in a real network, further tests were carried out for the MC2 measurement campaign. In that case, the CQI value on the side of the radio tester was not manually set. Therefore, depending on the estimated propagation parameters the adaptive selection of the modulation-code scheme for the communication link was chosen automatically based on the implemented internal algorithms in the emulated eNodeB.

Using the multivariate multiple regression for selected arguments of the function (2), the linear form of the quality metric function for the LTE RI for the KP2 scenario was determined as

(3) $$\begin{array}{c} BLER=f(RSRP_{b},RSRQ_{b},RSSI_{b},SINR_{b},MCS_{b})=\\ a+b\cdot RSRP_{b}+c\cdot RSRQ_{b}+d\cdot RSSI_{b}+e\cdot SINR_{b}+f\cdot MCS_{b}, \end{array}$$

where the coefficients of the function take the following values: *a* = 0.207, *b* = −0.718, *c* = −1.673, *d* = 0.937, *e* = −0.732, *f* = −0.102.

**Figure 4:**
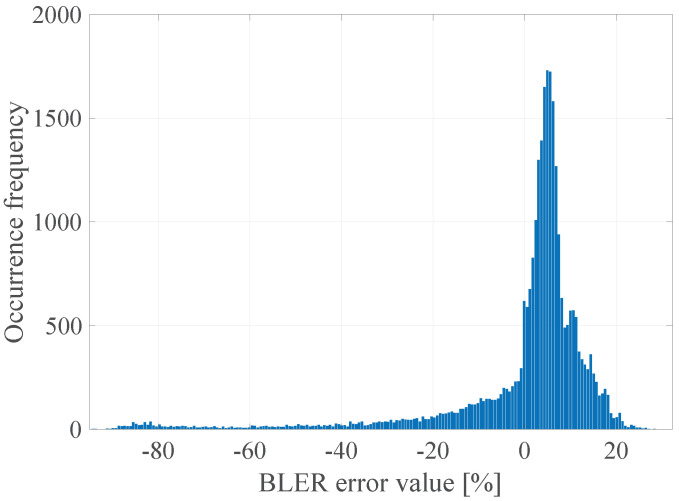
The histogram of the estimated errors of the BLER metric prediction for instantaneous radio parameters.

**Figure 5:**
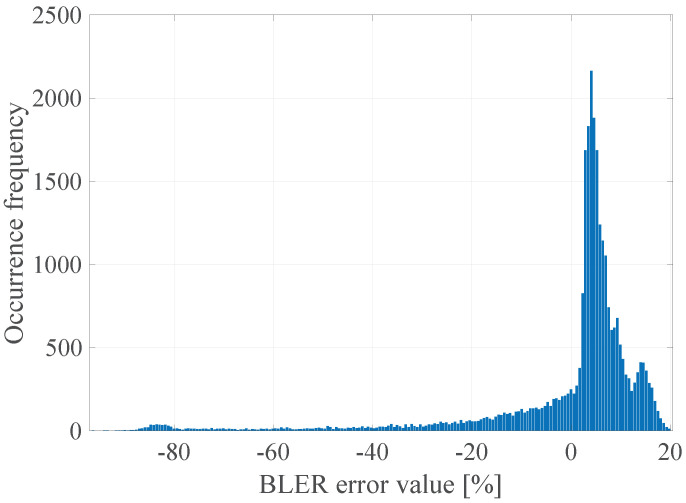
The histogram of the estimated BLER metric prediction errors for prediction using the 15th-order FIR filter.

**Figure 6:**
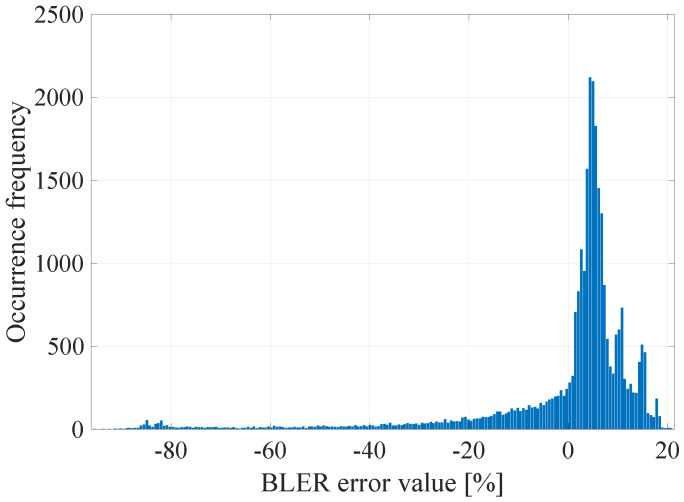
The histogram of the estimated BLER metric prediction errors for prediction using the median for the window length W=15.

**Figure 7:**
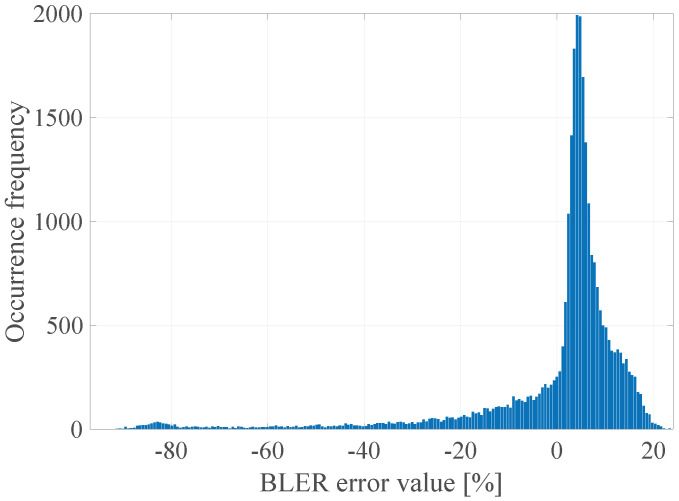
The histogram of the estimated errors of the BLER metric prediction for prediction with the use of separate linear models for each parameter for the window length W=15.

The GoF (Goodness of Fit) of the developed linear model with the empirical data was determined by calculating the determination coefficient $R^{2}$, which takes values in the range <0; 1>, where the value 1 means a perfect fit of the model to the empirical data [25]. $R^{2}$ was calculated for all the data and the model values that are consistent with the mathematical calculations of the model, but not with their physical interpretation (e.g., the BLER less than 0% or greater than 100%). The limitation of the values of the model was done after the actual regression had been performed. It was established that the value of the determination coefficient $R^{2}$ was 0.07, which means that, as expected, the linear model in this case does not accurately describe the measured radio parameters with the obtained error rate. It is also worth explaining that, in accordance to the form of the function (3), its arguments are the instantaneous values, not vectors of radio channel quality parameters as declared in (2). In general, it should be understood as the implementation of an additional operation in accordance with the selected radio channel prediction method, which were presented in the Section 6.1. Thus, the input data for the function (3) may be the instantaneous values (last vector elements) and the previously mentioned prediction with the use of the FIR filter with estimated length W=15. In addition, the prediction with the usage of the median for a window of length W=15 and prediction with the use of separate linear models of the parameters for a window of length *W* = 15 was also checked in that case. In the Figure 4, Figure 5, Figure 6 and Figure 7 the histograms of the estimated errors of the BLER metric prediction, defined as the difference of the actual value of the obtained BLER for the next transmission from the predicted value for the selected parameter prediction method in the used multivariate linear model, are shown.

The obtained results confirm that it is possible to determine the transmission quality with a given probability on the basis of the channel and network parameters measured before the transmission. The mean values of the errors for all the prediction methods were about 0%, and the error distributions showed the highest GoF to the Gaussian distribution with variable values of the RMSE (Root Mean Squared Error), i.e., 17.16%, 16.99%, 17.01%, and 17.02% respectively. Taking it into consideration, the lowest efficiency of the BLER metric prediction, as expected, was obtained for the method based solely on the instantaneous values of the radio parameters. The other three methods do not show significant differences for the analyzed MC2 measurement campaign, which in this case predestines the method based on the low-pass filtering to be used in the case of a multivariate linear model due to the computational complexity, which can be further reduced by designing the IIR (Infinite Impulse Response) filter.

However, as mentioned earlier, the determination of the BLER prediction error was done to estimate the low bound of the accepted solutions, and in the final solution the linear model should not be used due to the highly non-linear nature of changes in the radio parameters and their dependence on the estimated BLER in the physical layer.

## 7. Proposed Deep Learning Approach

Due to limited efficiency of the linear model, it is necessary to implement the non-linear function to predict the transmission quality metric, wherein the form of this function is unknown and generally 5-dimensional. Thus, it was decided that finding a multidimensional function that minimizes the transmission quality metric error can be effectively accomplished with the usage of the DL methods. In comparison to other methods, the proposed DL approach does not require direct extraction of the dataset features that will describe the raw data, but learning the efficient representation of the data by the feature decomposition, which leads to obtaining the complex transfer function out of simpler ones [26]. In that case, to obtain an efficiency gain, the learning process needs to be filled with a large number of data, however the already estimated model can be used even on a platform with low computational capabilities.

Contrary to the linear model, there is no justification for implementing the neural network separately for each parameter and then creation another network for analyzing the obtained output values. This may lead to a loss of information and relationship between the historical parameter values, which would potentially reduce the effectiveness of this method.

Undoubtedly, the problem of predicting the quality of the next transmission is a regression problem, and the obtained measurement data enable the implementation of the DL approach and the supervised learning phase. As a metric for the DL effectiveness, the MSE (Mean Squared Error) was chosen, however due to the interpretation of the results, in the article the RMSE metric is presented. This allowed minimizing the probability of a large difference between the predicted BLER value from the actual BLER value, by penalizing outlier values, which would negatively affect the final decision-making process of the DAA algorithm.

The inclusion of the historical data to make predictions in the next moment is a known issue, for example, to predict changes in the currency market and in the economy (the so-called time series prediction), in the video processing, or in general image analysis [27,28,29,30]. It has been proven that such a prediction can be performed in many ways with the use of several structures of the deep neural networks depending on the degree of their complexity, e.g., networks without feedback, convolutional networks, and recursive networks (including networks with cells). Bearing in mind that nodes of the distributed telemetry networks do not have high computing power and in general, it should be assumed that minimizing the energy consumption of such a device is one of the main goal that should be considered. Therefore, the network should be as low complex as possible and support the possibility of parallel computations in the greatest possible extent.

Taking into consideration the previously mentioned requirements and the possibility of implementing the target network, e.g., as a coprocessor in the structure of the FPGA (Field Programmable Gate Array) programmable matrix, to determine the form of the non-linear transmission quality function the architecture of the fully connected deep neural network FFNN (Feedforward Neural Network) was chosen [26]. Recursive networks are characterized by a sequential way of data processing, while convolutional networks significantly increase the number of computations necessary to make predictions. Due to the popularity of the FFNN and the numerous publications, where they are analyzed and described in detail, authors decided to not further describe this architecture and duplicate the common knowledge, however the description can be found e.g., in [26,31,32].

The optimal network architecture is generally unknown and despite the attempts to define rules, that can be found in the literature, they can only be used in the limited cases (e.g., for image processing). In the case of the proposed DL method, its hyperparameters should be determined in terms of:

- number of the input nodes;
- number of the hidden layers;
- number of nodes in the hidden layers (assuming a constant number of nodes in all layers);
- the form of the activation functions;
- learning rate.

Determining the network architecture hyperparameters is a complicated task and it is mainly solved with the use of [26] grid search method, genetic algorithms and Bayesian methods.

Taking into account the proposed architecture of the FFNN network and its relatively limited number of parameters to be checked, in relation to, for example, the convolutional networks processing images, it was decided to test their combinations without the use of additional algorithms searching the space of the possible solutions. Similar methodology was already used by the authors in [32]. Wherein, the search for the optimal hyperparameters was planned to be carried out in two stages in current research:

- preliminary grid search analysis and potentially coarse determination of the prediction efficiency dependence of the BLER as a function of the network architecture variable parameters;
- extended grid search analysis.

Based on the number of input nodes, the ranges of the hyperparameter variability and the learning parameters in the first stage were defined and presented in the Table 3.

The validation error rate is understood as the implementation of the counter, the value of which is incremented in the case of no reduction of the mean square error for the validation dataset. If the threshold value is reached, the learning process is terminated due to the lack of an increase in the learning efficiency in the next 20 iterations. The learning process was monitored with the use of the MSE metric and optimized with the use of the ADAM (Adaptive Moment Estimation) algorithm with the selected parameters, i.e., α=0.001, $\beta_{1}=0.9,\beta_{2}=0.999$ and $\epsilon =10^{-8}$ [26,32]. The ADAM algorithm is described in details in [33].

Moreover, the input nodes were arranged similar to the form of (3), where the vectors of the subsequent parameters were provided to the input layer of the FFNN. It should be mentioned that all the input data in the network were normalized to the range of values [0, 1] so that, despite the different possible ranges of the assumed values for all the radio parameters, the learning process could be implemented in the most effective manner. Additionally, due to the obvious different nature of the determining the non-linear function (2) method with respect to the linear function, the length of the sample history window was re-analyzed to determine the optimal value for the proposed DL model.

**Figure 8:**
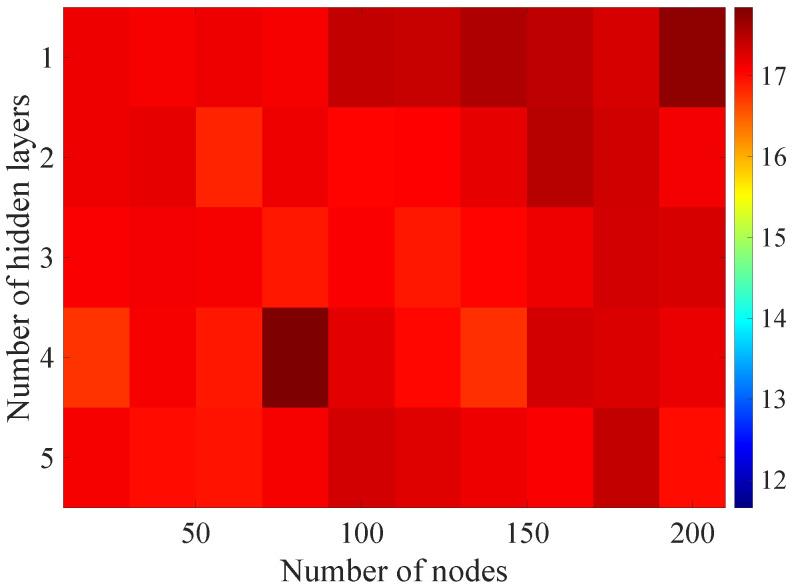
Matrix of the BLER RMSE metric predictions for the instantaneous values of radio parameters.

**Figure 9:**
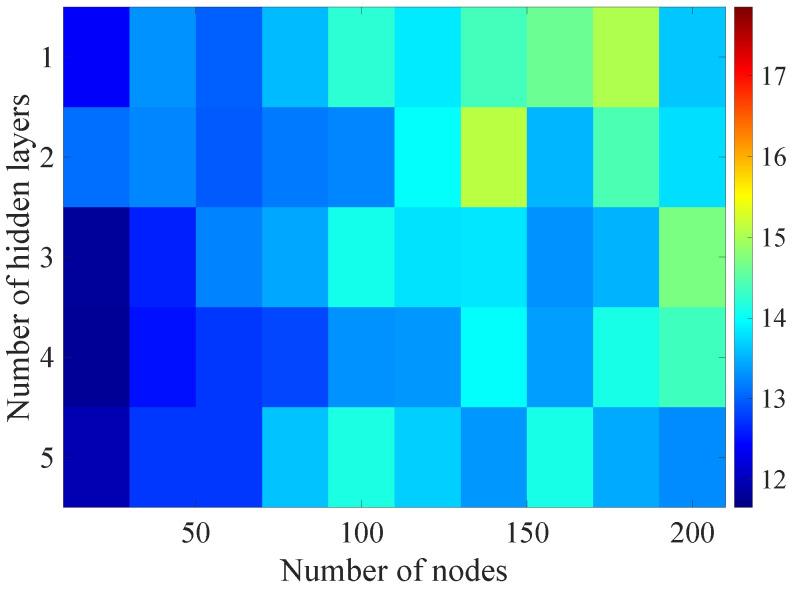
Matrix of the BLER RMSE metric predictions for the radio parameters history window W=5.

**Figure 10:**
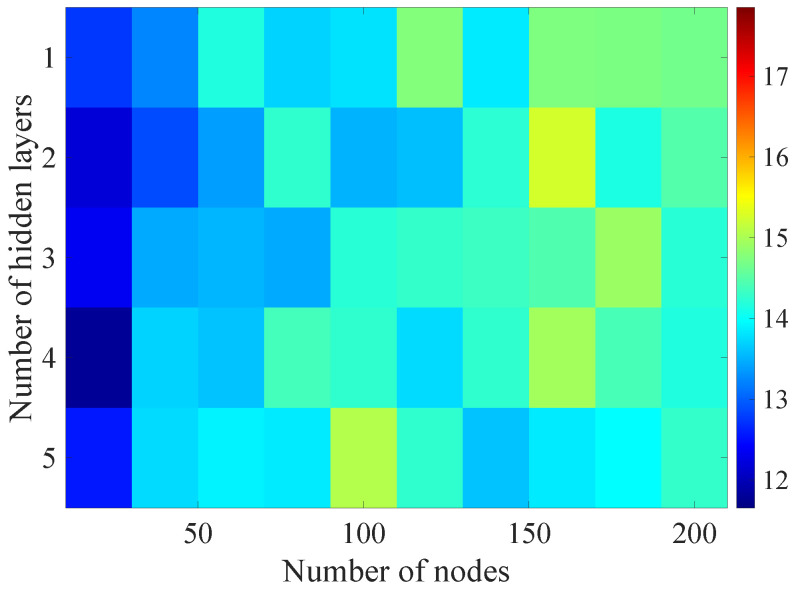
Matrix of the BLER RMSE metric predictions for the radio parameters history window W=10.

**Figure 11:**
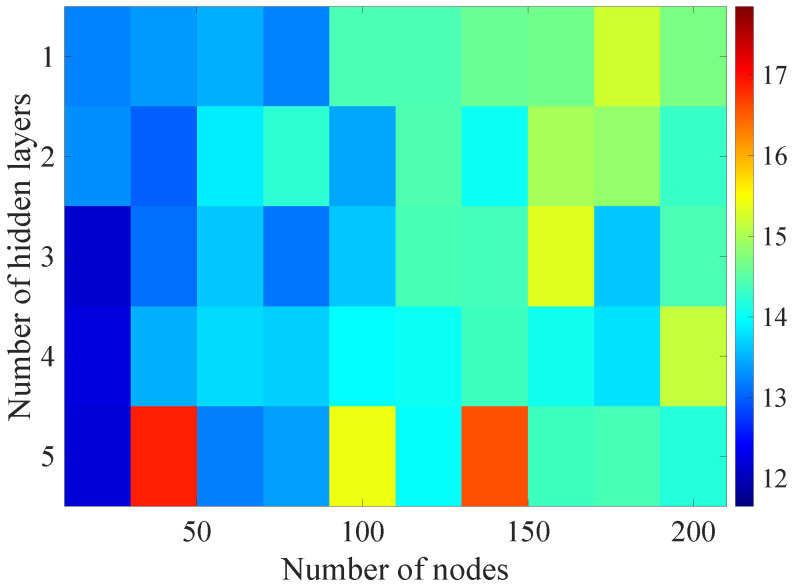
Matrix of the BLER RMSE metric predictions for the radio parameters history window W=15.

**Figure 12:**
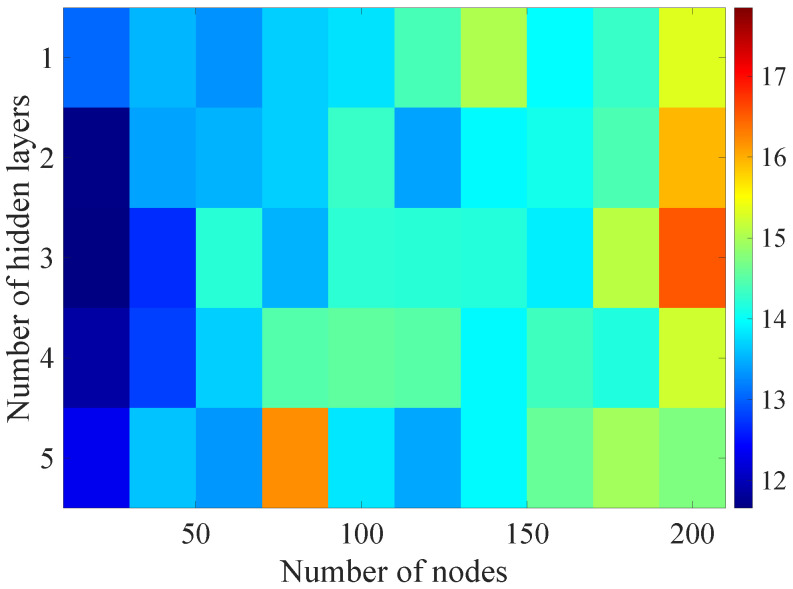
Matrix of the BLER RMSE metric predictions for the radio parameters history window W=20.

In the Figure 8, Figure 9, Figure 10, Figure 11 and Figure 12 graphically determined matrices of the RMSE BLER metric for selected values of the sample history window length are shown. In order to facilitate the analysis, the graphical scale of the RMSE representation was unified for all graphs.

On the basis of the presented results, it is possible to clearly determine the importance of the acquisition of the historical (previous) estimates of the radio parameters in relation to the implementation of the DL model based only on the instantaneous values of the radio parameters, regardless of the complexity of the model. For the instantaneous values the obtained RMSE values are in the range from 16.8% to 17.9%, with most models worse than the linear model for which the RMSE error value of 17.0% was achieved. This means that the DL models of the unidirectional network are characterized by a small gain compared to linear models (where the whole set of data was taken into the regression at one time), while for most of them, a much higher RMSE error regardless of the combination of the number of the hidden layers and nodes contained in these layers is obtained. The number of models that provide lower efficiency than linear model indicates that in that case, the DL approach is highly vulnerable for the learning data arrangement and initial weights, where no historical data can be used for improving the model convergence during the learning process.

As a part of the research, the activation function influence was also checked. However, the most efficient solutions were obtained for the tansig activation function, thus the further results are presented only for this type of function.

In the case of the DL models analysis for the selected set of sample history windows, all models are characterized by a lower mean square error value compared to the linear model and fall in the ranges from 11.7% to 16.9%, with the minimum value achieved for a 20-sample history radio parameters estimates. The first stage of the process of learning the grid search method shows that the reduction of the RMSE is strongly related to the smaller number of nodes in individual hidden layers, as indicated by the lowest values of RMSE errors as a function of the number of nodes presented in the Table 4.

The obtained results also indicate that increasing the number of the historic samples of the analyzed radio parameter estimates may increase the effectiveness of the DL models. In the Table 5 the obtained results for selected groups of the models defined in terms of the estimates history number along with the corresponding values of the effectiveness of these networks and the number of layers corresponding to the models is shown.

Taking into consideration the obtained results, selected parameters of the network architecture using the grid search method were verified and modified in the second stage of the learning process and presented in the Table 6.

Apart from increasing the number of hidden layers and the number of the model input nodes (related directly to the number of analyzed historical estimates of the radio parameters), the conditions determining the number of iterations in the learning process were also modified. On the basis of the obtained results and the average value of 1869 numbers of the iterations performed in the first stage, the validation error rate was increased.

**Figure 13:**
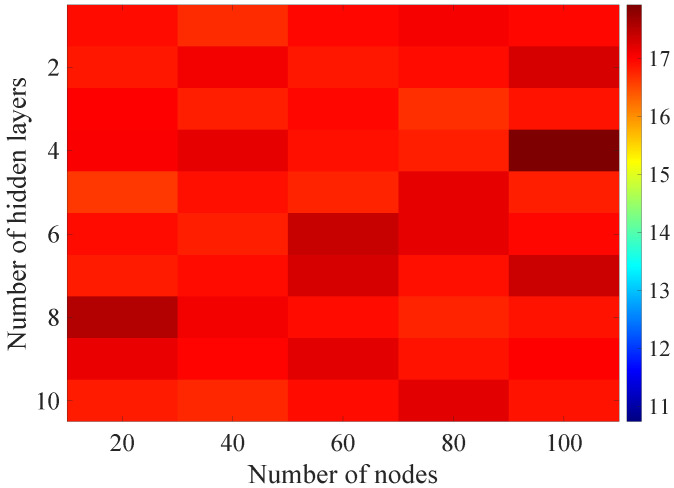
Matrix of the BLER RMSE metric predictions for the instantaneous values of the radio parameters in the second stage learning process.

**Figure 14:**
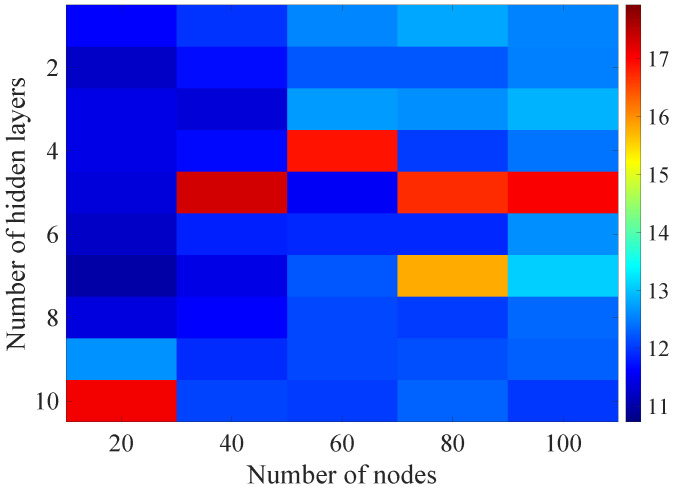
Matrix of the BLER RMSE metric predictions for the radio parameters history window W=5 in the second stage learning process.

**Figure 15:**
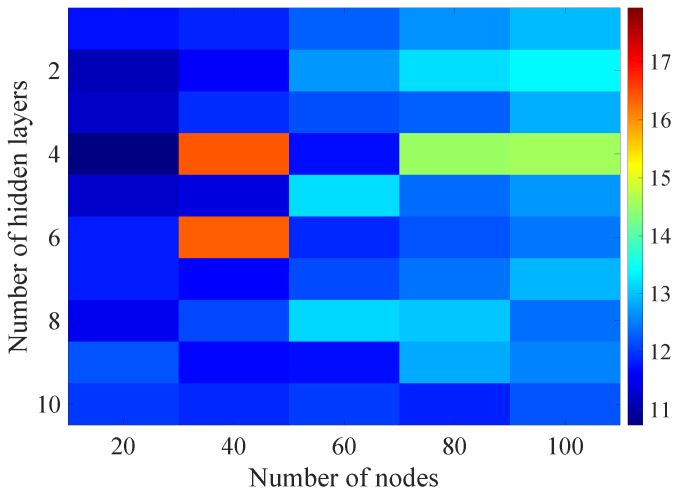
Matrix of the BLER RMSE metric predictions for the radio parameters history window W=10 in the second stage learning process.

**Figure 16:**
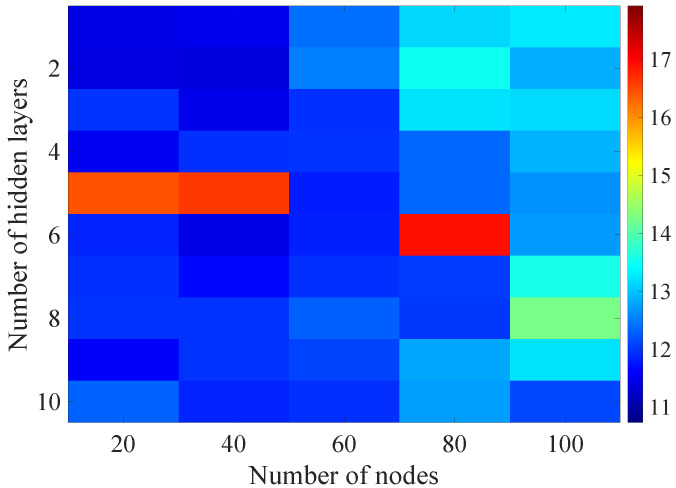
Matrix of the BLER RMSE metric predictions for the radio parameters history window W=15 in the second stage learning process.

**Figure 17:**
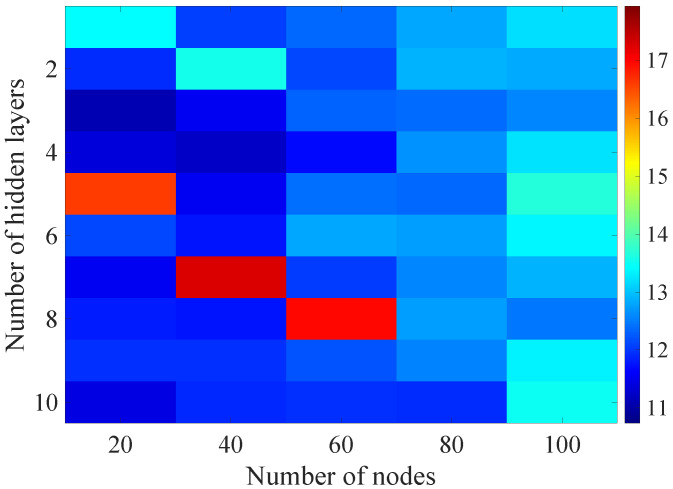
Matrix of the BLER RMSE metric predictions for the radio parameters history window W=20 in the second stage learning process.

**Figure 18:**
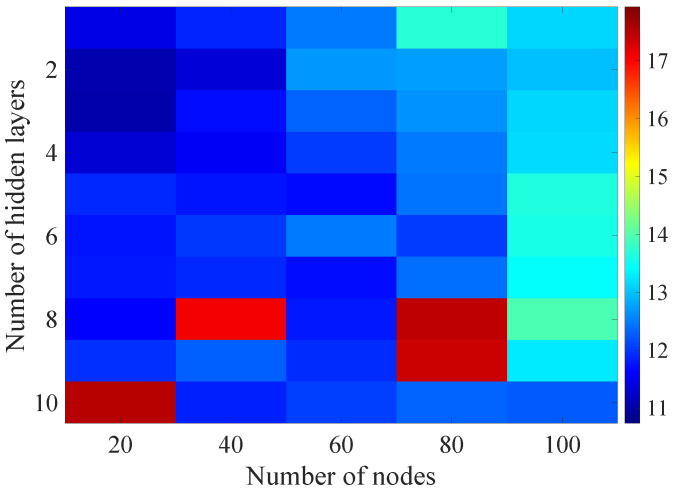
Matrix of the BLER RMSE metric predictions for the radio parameters history window W=25 in the second stage learning process.

**Figure 19:**
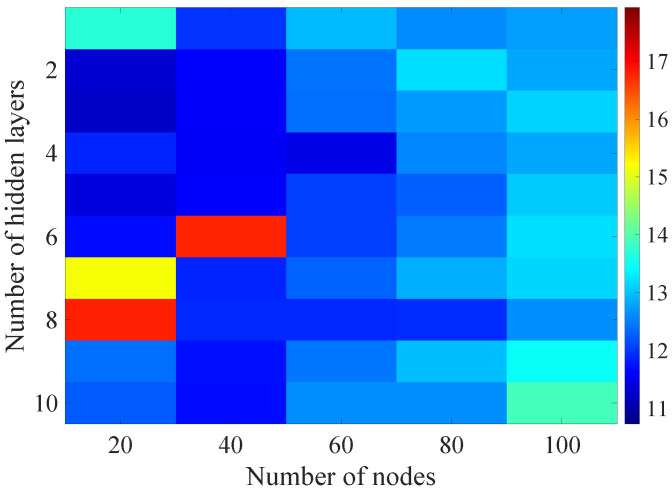
Matrix of the BLER RMSE metric predictions for the radio parameters history window W=30 in the second stage learning process.

In the Figure 13, Figure 14, Figure 15, Figure 16, Figure 17, Figure 18 and Figure 19 graphically determined matrices of the RMSE BLER metric for selected values of the sample history window length is shown.

Changes in the parameters of the learning process in the second stage significantly influenced the obtained results and the distribution of the error matrix for the individual scenarios of the analyzed windows of the history of the radio parameter estimates in relation to the first stage for the cases where the historical values of the radio parameter estimates were analyzed. As in the first stage, the effectiveness of the DL models is significantly lower, i.e., are in a range from 16.6% to 17.9%, when analyzing only the instantaneous values, which means that the range of the possible values was wider than in the first stage of the learning for the initial parameters of the learning process.

For models that take into account historical estimates, the error matrix values range from 10.7% to 17.5%. This means that modification of the parameters of the learning process brought positive effects in the form of reducing the obtained RMSE with about 15% profit comparing to the most effective model from the first stage. However, attention should also be paid to the occurrence of the architectures reaching error values greater than the linear model that failed to converge due to the threshold of the validation error. This means that these models strongly depended on the distribution of the training data and the initialized weight values.

In the Table 7 the calculated values of the RMSE BLER metric for selected numbers of the analyzed historical radio parameters are presented.

On the basis of the obtained results, it can be determined that increasing the number of nodes and the number of layers in general did not allow increasing the accuracy of the BLER metric prediction during the next transmission. The best results were obtained mostly for the number of nodes in hidden layers equal to 20 and a different number of the hidden layers. However, it should be noted that the inverse proportional dependence between the number of hidden layers and the number of historical samples in terms of the obtained RMSE is clearly visible. The increase of the historical data memory allows using models with the less number of hidden layers while maintaining a comparable RMSE value for each scenario. The differences between the individual models are not greater than the 0.7% RMSE error relative to the most effective model. For the analyzed error matrices, 2% of the obtained models have the final RMSE error values of the BLER higher than the linear model, while 34% of the obtained models are characterized by more than a double profit compared to the linear model. This indicates the significant increase in the efficiency of the BLER metric prediction using the DL and the high convergence of the developed models for the defined problem.

**Figure 20:**
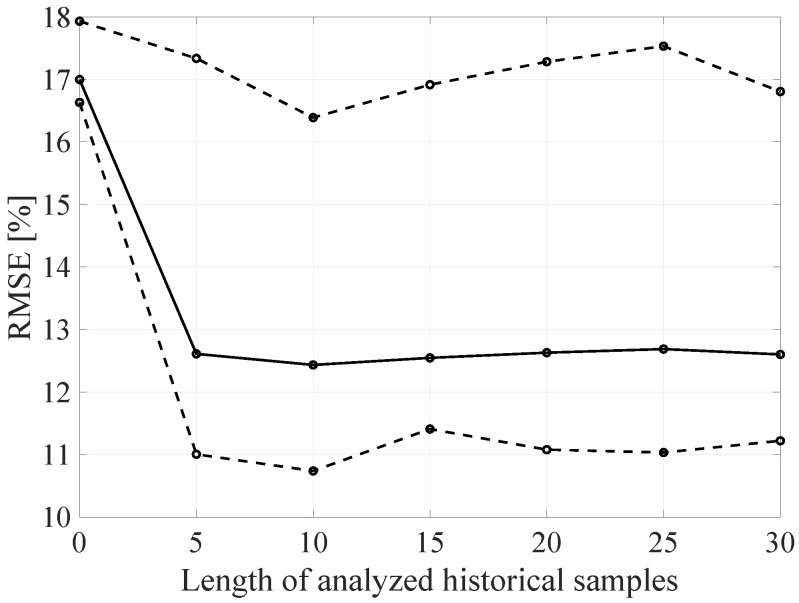
The RMSE BLER as a function of the analyzed time window length (number of the input parameters).

**Figure 21:**
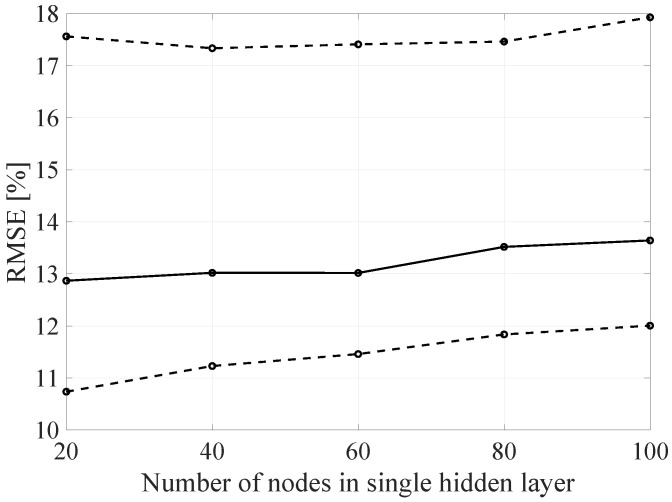
The RMSE BLER as a function of the node number in the hidden layer.

**Figure 22:**
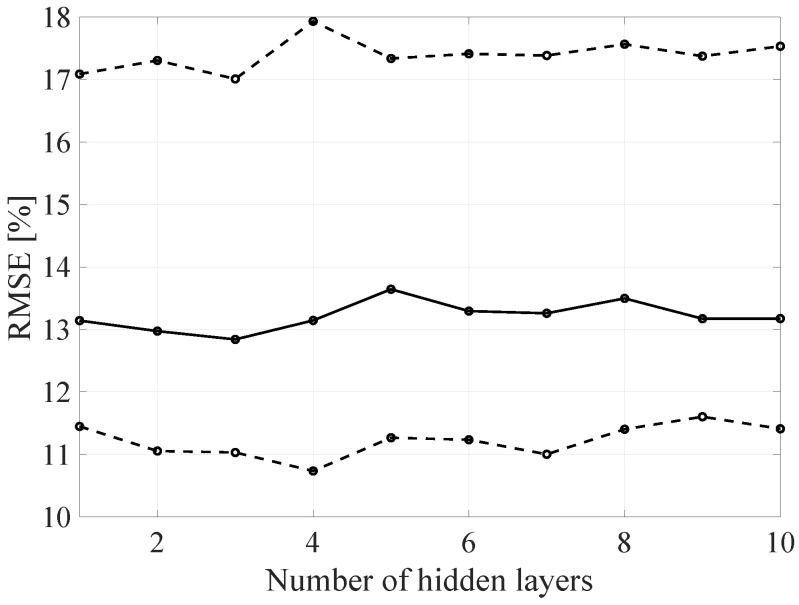
The RMSE BLER as a function of the hidden layers number.

In order to analyze it more precisely and to verify the influence of the individual network architecture parameters on the resulting RMSE, the architecture of the proposed DL method was decomposed by varying the selected architecture parameter with constant values of the other parameters. In the Figure 20, Figure 21 and Figure 22 the BLER RMSE as a function of selected parameters of the FFNN network architecture with marked minimum and maximum boundary values and average values are presented.

As mentioned, changes in the parameters of the learning process in the second stage significantly influenced the obtained results and the distribution of the error matrix for individual configurations of the FFNN network architecture. The BLER root mean square errors as a function of the analyzed time window length indicate that increasing the sample history above 5, does not increase the efficiency of the given models, but increases the number of the calculations needed to be performed for the prediction of the transmission quality metric estimate. As in the case of the first stage of the learning process, increasing the number of nodes in the hidden layers resulted in a proportional (linear) increase in both the minimum and average RMSE for the analyzed architectures. By analyzing the number of hidden layers, it can be determined that the lowest value was obtained for the number of four hidden layers, but in this case it is not possible to determine the unambiguous trend of changes and the impact of the number of hidden layers on the efficiency of the network.

Based on the obtained results, the architecture for the RMSE of the BLER, i.e., 10.7%, was determinedand its parameters are presented in the Table 8.

**Figure 23:**
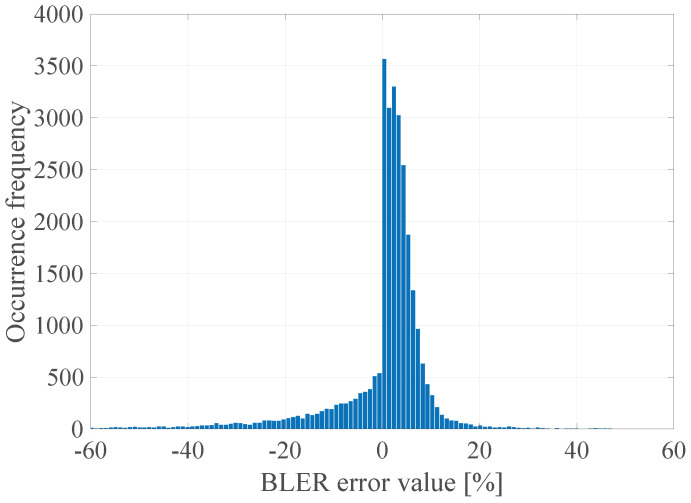
The BLER error histogram of the proposed DL model.

In the Figure 23 the histogram of the BLER error value for the determined DL model is presented.

The obtained results confirm that it is possible to determine the transmission quality with a given probability on the basis of the channel and network parameters measured before the transmission using the proposed DL method. The non-linear form of the function (2) estimated with the use of the determined FFNN model is characterized by the zero mean value of the prediction error, and the error distribution shows the highest GoF to the Gaussian distribution with a 10.7% RMSE, which is a significant gain (almost 40%) in relation to the linear model (3) with the lowest RMSE 17.01%.

To sum up, the non-linear relationship between link parameters measured by LTE modem and transmission efficiency measured by BLER level was determined as a model of the deep neural network. It enables the determination of the BLER value with a greater accuracy in relation to the lower bound estimated by the multidimensional linear function. The obtained results also confirm the validity of using the DL method to predict the transmission quality metric of the LTE RI for selected radio parameters and can be effectively used in the data allocation algorithm in the heterogeneous nodes. The analysis of the history of measurements significantly improves this process, however, after exceeding the limit value, the obtained estimates do not increase the efficiency of the model due to the lack of correlation with the current state of the radio channel.

## 8. Conclusions

The communication heterogeneity is usually a solution for increasing the reliability as well as the capacity of the network. The urban environment is treated as harsh in the context of the radio propagation conditions. The coexistence of the same types of networks causes a significant channel quality degradation, usually of a stochastic character, which further implies the usage of algorithms and methods for monitoring and managing the data streams.

As a solution, the novel DL-based channel state estimation method for the data allocation algorithm was proposed. It is especially designed for the heterogenous telemetric devices equipped with the LTE RI. The presented results clearly show that it is possible to estimate the transmission quality on the basis of the channel and network parameters measured before the transmission by a commercial radio module. The obtained prediction effectiveness, the RMSE below 11% of the BLER, satisfies the requirements for providing high reliability communication services, especially for the critical infrastructure. The proposed algorithm is based on the DL that allowed determining the non-linear dependence between the measured radio channel parameters and the transmission effectiveness expressed as BLER. In the paper the methodology of the proper analysis is also highlighted, showing the influence of the historic data on the estimation quality. It was proven that the inverse proportional dependence between the number of hidden layers and the number of historical samples in terms of the obtained RMSE exists. The increase of the historical data memory allows using models with the less number of hidden layers while maintaining a comparable RMSE value for each scenario.

It should be noted that the proposed approach can be easily used in parallel computing solutions and node architectures, where the computational complexity of the already trained model is low. Thus, it can be implemented in the practical applications in the heterogeneous telemetry devices, even based on the microcontrollers.

During the future works, the analysis of other RIs commonly used in the modern heterogeneous terminals, especially the NB-IoT, should be considered. In addition, the analysis of the activation tansig function mapping accuracy (its resolution) can be also investigated.

## Figures and Tables

**Table 1:** Emulated base station operating parameters for the MC1 measurement campaign.

Parameter	Value	
Frequency band	OB1	UL: 1920–1980 MHz
		DL: 2110–2170 MHz
Bandwidth	10 MHz	
Fading profile	EP5 Medium	
Doppler shift	1 Hz	
SNR range	from −3 dB to 30 dB every 1 dB	
Transmitted signal power spectral density	−75 dBm/15 kHz	
Number of subframes transmitted within a single transmission	100	
CQI (Channel Quality Indicator)	{1, 3, 5, 7, 9, 11}	
Measurement interval	max. 300 ms	

**Table 2:** The obtained results of the Pearson correlation coefficient for the MC1.

CQI	${\mathrm{RSRP}}_{b}$			${\mathrm{RSRQ}}_{b}$			${\mathrm{RSSI}}_{b}$			${\mathrm{SINR}}_{b}$		
	μ	M	All	μ	M	All	μ	M	All	μ	M	All
1	−0.4	−0.4	−0.4	−0.4	−0.4	−0.5	−0.4	−0.4	−0.3	−0.4	−0.4	−0.4
3	−0.5	−0.5	−0.4	−0.5	−0.5	−0.6	−0.4	−0.4	−0.4	−0.4	−0.4	−0.4
5	−0.6	−0.6	−0.7	−0.7	−0.7	−0.8	−0.5	−0.5	−0.6	−0.6	−0.6	−0.6
7	−0.7	−0.7	−0.8	−0.7	−0.7	−0.9	−0.6	−0.6	−0.7	−0.6	−0.6	−0.8
9	−0.7	−0.7	−0.9	−0.5	−0.5	−0.8	−0.6	−0.6	−0.8	−0.6	−0.6	−0.8
11	−0.7	−0.7	−0.9	−0.4	−0.4	−0.7	−0.6	−0.6	−0.9	−0.6	−0.6	−0.8

**Table 3:** The hyperparameter variability and the learning parameters in the first stage of the learning process.

Parameter	Value
Number of hidden layers	from 1 to 5
Number of nodes in the hidden layers	from 20 to 200 with the step of 20
Activation function	tansig, ReLU, tanh, sigmoid
Number of the input nodes	5 parameters and their multiple depending on the length of the analyzed history, i.e., from 0 to 20;
Learning rate	0.001
Number of the learning iterations	max. 5000 or max. 20 validation error rate
Dataset split	60% learning
	20% validation
	20% testing

**Table 4:** Calculated the smallest RMSE for selected numbers of nodes in hidden layers.

Number of nodes	20	40	60	80	100	120	140	160	180	200
RMSE [%]	11.7	12.5	12.7	12.9	13.3	13.3	13.3	13.3	13.5	13.3

**Table 5:** Calculated the smallest RMSE for the selected numbers of nodes in hidden layers.

Number of the history samples	5	10	15	20
RMSE [%]	11.8	11.78	12.17	11.66
Number of the hidden layers	4	4	5	3

**Table 6:** The hyperparameter variability and the learning parameters in the second stage of the learning process.

Parameter	Value
Number of hidden layers	from 1 to 10
Number of nodes in the hidden layers	from 20 to 100 with the step of 20
Activation function	tansig
Number of the input nodes	5 parameters and their multiple depending on the length of the analyzed history, i.e., from 0 to 30;
Learning rate	0.001
Number of the learning iterations	max. 10,000 or max. 50 validation error rate
Dataset split	60% learning
	20% validation
	20% testing

**Table 7:** Calculated the smallest RMSE for the selected numbers of the analyzed historical radio parameters.

Number of the historical samples	5	10	15	20	25	30
RMSE [%]	11.0	10.7	11.4	11.1	11.0	11.2
Number of the hidden layers	7	4	2	3	3	3
Number of the nodes	20	20	40	20	20	20

**Table 8:** The architecture parameters of the chosen DL approach.

Parameter	Value
Number of hidden layers	4
Number of nodes in the hidden layers	20
Activation function	tansig
Number of the input nodes	55

## Data Availability

Datasets are available for free from the authors with the CC BY license.
